# The State of Diabetes in Kansas: A Community Centered Approach to the Treatment of Diverse Populations

**Published:** 2017-11-30

**Authors:** Tiffany E. Schwasinger-Schmidt, Jon P. Schrage, Justin B. Moore, Betty M. Drees

**Affiliations:** 1 University of Kansas School of Medicine-Wichita, Department of Internal Medicine, Wichita, KS; 2 Double Arrow Metabolism, LLC, Wichita, KS; 3 University of Missouri-Kansas City School of Medicine, Kansas City, MO

**Keywords:** prediabetes, diabetes mellitus, food safety, community outreach

## Background

Diabetes mellitus continues to have a significant negative impact on the overall health of Americans. Current estimates of the prevalence of diabetes indicate that approximately 29 million Americans have diabetes, which is equivalent to 9% of the population.^1^,^2^ Additionally, 28% of this population is undiagnosed, and therefore untreated. When the data are broken down by state, approximately 9.5% of Kansans have diabetes with the prevalence increasing over time.^1^,^2^ A comparison between states reveals that Kansas ranks 22^nd^ in the total number of diagnosed cases of diabetes mellitus.^1^,^2^

The prevalence of diabetes is associated with socioeconomic factors including income level, education, ethnicity, and geographic location with regards to rural or urban dwelling.^1^–^3^ In Kansas, approximately 11% of adults with an average annual household income of less than $50,000 per year have diabetes, as compared to 6% in households earning more than $50,000 per year.^3^ The incidence of diabetes is higher in people without a college degree at 9.7%, compared to those with a college education at 6.4%.^1^,^2^ Data of age-adjusted prevalence of diabetes indicate that the number of diagnosed cases are higher among Non-Hispanic African Americans (13.2%), followed by Hispanics (12.8%), Asians (9%), and Non-Hispanic Whites (7.6%).^1^,^2^ The greatest prevalence of diabetes among ethnic groups is found in Native Americans at 15.9%.^1^,^2^

Estimates of pre-diabetes prevalence, defined by an HbA1c of 5.7 – 6.4%, or fasting plasma glucose of 100 – 125 mg/dL, indicate that approximately 37% of the population age 20 and older have pre-diabetes, which equates to 86 million Americans.^1^ Further review of this data indicates that 51% of the population with pre-diabetes are older than age 65.^1^,^4^ Within this population, approximately 90% are unaware of their diagnosis.^5^ Age-adjusted data reveal that prevalence among ethnic groups is similar for Non-Hispanic Whites (35%), Non-Hispanic African Americans (39%), and Hispanics (38%).^1^ Approximately 15 – 30% of people with pre-diabetes will develop diabetes within five years without treatment, with 70% developing diabetes at some point during their lifetime.^1^,^4^–^7^

A comparison of diabetes prevalence by demographic location in Kansas reveals similar numbers of people with diabetes in urban populations (11.8%) as compared to rural areas (12.7%).^3^ Analysis of the number of people with pre-diabetes showed similar rates among urban (3.7%) and rural (3.1%) locations.^1^,^2^ Socioeconomic factors of lower education and income were associated with increased incidence of pre-diabetes with the highest rates noted in rural Hispanics (19.3%) and urban African Americans (22.9%).^2^,^3^

## Diabetes Impact on Health

Diabetes has a significant impact on health with high rates of associated morbidity and mortality.^5^,^8^ Diabetes is the 7^th^ leading cause of death in both the United States and Kansas according to death certificate data, which is likely to be an underrepresentation of the true incidence of diabetes related deaths.^3^,^4^ This disease is known to double the risk of death from any cause, and additionally results in a 2 to 4 fold increase in the risk of death from cardiovascular disease and stroke.^1^,^4^,^8^ The risk of myocardial infarction (MI) in patients with diabetes mellitus is equivalent to the risk in non-diabetic patients with a prior MI, causing it to be considered a coronary artery disease risk equivalent.^8^ In 2014, more than 14% of Kansans who have diabetes were diagnosed with a stroke or coronary artery disease, with 14.2% of this population having an MI within that year.^3^ This is compared with 3% of the population without diabetes having an acute myocardial infarction.

Diabetic nephropathy is the leading cause of renal failure in the United States. In 2011, approximately 50,000 people began treatment for chronic kidney disease due to diabetes.^1^,^8^ Population estimates of the prevalence of renal failure due to diabetes indicate that at least 229,000 people in the United States are on dialysis or have a kidney transplant.^1^ Statistics from Kansas in 2014 indicated that 9.7% of patients who have diabetes have chronic kidney disease.^3^ Diabetes also is the leading cause of blindness and the cause of more than 10,000 new cases of blindness in the United States each year.^1^,^4^,^8^ Diabetic retinopathy affects approximately 16% of Kansans who are diagnosed with diabetes.^3^ Approximately 60% of non-traumatic lower extremity amputations are due to diabetes with resultant increases in morbidity and mortality due to infection.^4^

## Economic Impact of Diabetes

The economic impact of diabetes is profound, with costs associated with a diagnosis of diabetes mellitus estimated at $245 billion nationally for both health care associated costs and costs associated with reduced productivity.^1^,^5^ Estimates of cost associated with patients that have not yet been diagnosed with diabetes or pre-diabetes total an additional $25 billion.^1^,^5^,^6^ When comparing the average cost of care between people with and without diabetes, the cost for total health care spending of people who have diabetes is over twice the cost for a patient without diabetes.^1^,^5^ The treatment of diabetes in Kansas costs an estimated 2.6 billion dollars in both direct and indirect costs.^3^

## Challenges in the Treatment of Diabetes

Due to the increasing prevalence of diabetes, the majority of treatment is occurring in primary care offices around the country.^1^,^4^ Kansas is no exception, due to the limited number of endocrinologists, who are located mainly in the major cities of Kansas. A recent focus group facilitated by the University of Kansas Medical Center surveyed primary care physicians across the state to identify barriers to the treatment of diabetes. 9 This survey found that physicians cited training and support in the diagnosis and management of diabetes as their primary need.

The United States Preventive Services Task Force has recommended screening for abnormal blood glucose levels in primary care settings for adults ages 40 – 70 who are overweight or obese, which includes approximately 66% of the American population.^4^,^6^ Despite this recommendation, current estimates indicate that approximately 20 – 30% of the population remains undiagnosed.^1^,^2^,^5^ Data regarding the detection and treatment of pre-diabetes in primary care settings is even more concerning in that the diagnosis often is missed, with only 25% of patients receiving treatment with lifestyle modification counseling.^10^,^11^ National estimates of in-patient hospital costs for patients with diabetes in 2001 indicated that approximately 66% of admissions could have been prevented by improved outpatient care and monitoring.^6^

This data highlights the need for improved diabetes care provided by primary care physicians and the need for a partnership with community resources. Diabetes cannot be treated in isolation and requires a team approach that includes physicians, nurse practitioners, physician assistants, nurses, diabetes educators, registered dieticians, fitness facilities, weight loss organizations, local agricultural resources, community leaders, local and state government representatives, and validated online tools and resources to help patients understand the disease and develop skills to promote overall health. This article presents different treatment modalities available to patients and physicians in different geographic settings that target diverse patient populations.

## Urban Community Resources

### National Diabetes Prevention Program

The YMCA of the USA, via a Health Care Innovation Award from the Centers for Medicare and Medicaid Services, developed a Diabetes Prevention Program delivered in regional networks of participating YMCAs nationwide.^12^ This program operates with the goal of preventing the development of diabetes through a reduction in dietary fat, education on locus of control, and increased physical activity. The program utilizes a trained lifestyle coach to facilitate small group discussions about ways to improve the overall health of participants. Participants of the program attend 25, one-hour sessions over the course of a year with an end goal of 5 – 7% reduction in body weight and increased physical activity levels of at least 150 minutes per week. Participants are taught about healthy eating and ways to limit portion size. They additionally explore barriers to weight loss and healthy living in small group discussions. This fosters not only a sense of unity among the participants, but also helps participants learn from each other on ways to overcome challenges with healthy living and to identify personal barriers.

Results obtained from participants in the YMCA Diabetes Prevention Program (DPP) indicate that involvement in the program can result in a 60% reduction in the number of people who have pre-diabetes developing overt diabetes mellitus.^12^,^13^ Qualification for the program includes age greater than 18, overweight with a body mass index (BMI) greater than 25, and a diagnosis of pre-diabetes by a physician. The cost associated with the program is $429.00, adjustable by income, which includes a three-month YMCA family membership. Income-based pricing and scholarships are available to participants that qualify. Modeling of initial results of the YMCA DPP program indicated that, if expanded nationally, it would prevent or delay approximately 885,000 cases of type 2 diabetes mellitus in the United States and produce savings of $5.7 billion.^12^,^13^ The success of the YMCA’s program led to the development of the National Diabetes Prevention Program (https://www.cdc.gov/diabetes/prevention/index.html), through which any organization can adapt the curriculum and achieve Diabetes Prevention Program recognition through the United States Centers for Disease Control and Prevention (CDC). Such recognition will allow an organization to bill Medicare for a DPP beginning January 1, 2018 (https://innovation.cms.gov/initiatives/medicare-diabetes-prevention-program/).

Clinics across Kansas can partner with local Diabetes Prevention Programs, either through the YMCA or other organizations (an index of CDC-recognized programs can be found at https://www.cdc.gov/diabetes/prevention/index.html). Through this partnership, patients receive the benefits of access to a fitness facility, education about healthy eating, support from a community of people with pre-diabetes, and education about the benefits of weight loss and overall health promotion. Providers can help patients obtain the community support they need to improve their overall health and additionally receive updates from the DPP program about the patient’s progress in the program to provide additional support and encouragement.

The University of Kansas Center for Internal Medicine in Wichita, Kansas has partnered with the local YMCA to refer patients into the YMCA DPP. Between July 2015 and March 2016, 261 patients within the clinic were screened with a HbA1c. Of those screened, 104 patients met the criteria and were given information about the YMCA DPP. A total of 38 patients were referred to the program with 12 patients enrolled. The average initial weight of our patient population entering the program was 252 pounds with a BMI of 41. Average initial HbA1c of participants referred to the program was 6%. Patients attended on average 74% of the classes over the duration of the one-year program. Through the YMCA DPP, patients lost an average of 4% of their initial body weight. HbA1c values at the completion of the program were unable to be attained as some patients were lost to follow-up. The results obtained from our study illustrated the importance of programs like the YMCA DPP, which result in significant reductions in the development of diabetes mellitus and improved overall health in our clinic population.

### Diabetes Self-Management Program

The Diabetes Self-Management Program (DSMP) is a workshop for patients with type 2 diabetes mellitus that is facilitated by two trained peer leaders, one or both of whom have diabetes themselves. Participants attend for 2.5 hours per week for six weeks in groups of 12 – 16 in community settings such as churches, community centers, libraries, and clinics and work through a detailed manual regarding diabetes management. A randomized, controlled trial completed in 2008 showed that six months after the workshop, participants had significant improvements in patient activation and self-efficacy, along with significant improvements in depression, symptoms of hypoglycemia, communication with physicians, healthy eating, and the ability to read food labels; most of which persisted at 12 months.^14^ There were no significant changes in health care utilization or HbA1c levels, though it should be noted that HbA1c values were already in the desirable range at the beginning of the study for most participants. In Wichita, the DSMP is offered through Wichita State University’s Community Engagement Institute (http://communityengagementinstitute.org/).

### Elimination of Food Deserts

Recent changes in the economy have led to the unfortunate loss of many businesses throughout communities. This loss has the most profound impact on health when it results in the elimination of community grocery stores, leading to reduced access to food. This can create “food deserts” where people that were initially able to walk to the grocery store located within their community have significant challenges in being able to obtain not only healthy food, but often being able to obtain food in general.^15^

One way that practitioners can aid in the elimination of food deserts is to work with local community leaders to find ways to provide healthy food to citizens. By working with the local government representatives, public transportation might be rerouted to ensure access to a grocery store. By altering bus routes, people are able to arrive at their destinations, but have the opportunity to obtain healthy food while traveling between work and home.

In Sedgwick County, the Health and Wellness Coalition of Wichita performed an assessment in 2013 revealing the presence of 44 square miles of food desert in Wichita (http://ctb.ku.edu/sites/default/files/chapter_files/wichita_food_desert_study.pdf). The assessment was followed by the “Behaviors Behind Limited Food Access” report showing that 25% of people in Sedgwick County lacked access to healthy foods, and that their access was limited by cost, quality and quantity of available food, lack of transportation, poor store quality and characteristics, poor sources of food outside grocery stores, and a lack of personal cooking skills (https://hwcwichita.org/content/upload/files/The%20Hurdles%20to%20Healthy%20Food%20Access.pdf). The Sedgwick County local food assessment, completed in 2015, revealed that if local policies encouraged growers to provide only 5% of the fruits and vegetables available to consumers in grocery stores in Sedgwick County, it could lead to a local economic impact of $54.6 million (https://hwcwichita.org/content/upload/files/Food%20Systems%20Assessment%20Report%20-%20December%202015.pdf). This led to the formation of a local Food Policy Committee to work with community leaders to promote local ownership of the food supply, including strategies such as neighborhood and community gardens and deregulation of the formation of local farmers markets. By working with local farmers, community leaders can identify areas in need of access to food. This cooperation allows citizens a healthy option to obtain food and additionally helps promote local famers and locally grown produce.

### Community Exercise

A frequent challenge to health in urban areas is finding a safe place to exercise within the community. Unfortunately, many people live in areas that are not safe to walk due to challenges with infrastructure or due to local neighborhood dynamics. One way to help with this challenge is to work with local community centers to allow patients to have a safe area to walk or exercise. Many fitness facilities, including the YMCA, offer income-based pricing that can provide patients access to a safe place for exercise at a reduced cost of membership. Patients also should be encouraged to utilize air-conditioned and heated sites for exercise, including walking the mall or local retail stores to get exercise in a comfortable environment.

Additional ways that practitioners can help patients attain safe walking places in their neighborhoods are to work with local government officials to construct sidewalks in areas throughout the community. Promotion of bike paths or local walking trails can provide a safe way for people in the community to exercise. Practitioners can work with local government representatives to ensure adequate lighting and placement of emergency contact stations to alert local law enforcement officials if needed. Adults aged 30 – 64 in 8,777 neighborhoods in Southern Ontario cities (London, Ottawa, Toronto, Hamilton) were roughly 20% less likely than their peers between 2001 and 2012 to be diagnosed with type 2 diabetes if they resided in a neighborhood with a walkability index in the top quintile compared to the lowest quintile of walkability.^16^

Community organizations and churches play an essential role in the promotion of health and disease prevention by advocating healthy eating and group exercise. Practitioners that are members of these organizations play a unique role in community engagement by organizing group exercise through walks/runs to benefit charitable organizations or walking groups for socialization. In addition, these community gatherings can provide an avenue to promote health and distribute information about healthy lifestyle changes. An example of an innovative strategy in Wichita is the development of a Joint Use Agreement between Botanica (www.botanica.com), the local botanical gardens, and Health ICT (www.healthict.org), a CDC-funded organization devoted to reducing the incidence of type 2 diabetes mellitus, by which employees of certain companies can gain admittance to the botanical gardens for $1 for exercise purposes.

### Weight Loss Programs

Lifestyle intervention is the cornerstone of diabetes and pre-diabetes treatment through reduced caloric intake and moderate exercise to promote weight loss in overweight and obese patients.^17^,^18^ Weight loss programs are a viable treatment modality for not only the prevention of pre-diabetes progression to diabetes mellitus, but also to improve blood sugar levels in known diabetics. Most urban areas have dedicated weight loss centers that utilize proprietary meal replacements for weight loss. Financially, this can be a challenge for patients, but by adding up the cost of medications, herbal supplements, physician office visits, and other weight loss modalities that patients are using at home, it may be more cost effective to engage in an organized program that combines education about eating habits, food quality, exercise, and behavioral modification.

There are numerous proprietary weight loss programs available to patients. A recent meta-analysis showed that significant weight loss has been observed in patients adhering to a low carbohydrate and low-fat diet; however, it is not necessarily the composition of the diet that determines weight loss, but patient adherence to the diet that has the most profound impact on weight loss and overall health.^19^ Long-term weight loss is best achieved through programs that combine healthy eating and exercise with behavioral modification therapy.^17^

Medications can help patients augment weight loss and improve overall health.^20^ These medications must be used as an adjunct to healthy lifestyle interventions for highest efficacy and maintenance of weight loss. The challenge that most providers face with medication interventions is that patients often can rely on the medications without implementation of healthy lifestyles, thereby regaining the weight lost or gaining additional weight once the medications are discontinued.^21^

### Community Outreach

Community engagement in health and prevention of chronic disease is a cornerstone of any medical practice. Raising community awareness can be achieved by establishing community diabetes screening programs utilizing local clinics, hospitals, medical schools, the health department, or community organizations including local chapters of the American Diabetes Association. At these locations, patients receive low cost or no cost screening of HbA1c’s and obtain additional information or referrals for further interventions and treatments if diagnosed with pre-diabetes or diabetes mellitus. HbA1c testing in urban clinics and community outreach health centers allows for greater patient population screening and identification of a substantial number of patients with undiagnosed pre-diabetes and diabetes mellitus that requires treatment.^22^

These outreach clinics can be a means to disseminate health information to lower socioeconomic populations about overall health and health promotion.^14^,^22^,^23^ The location of community outreach efforts needs to be centered in areas where patients have significant barriers to care and areas where transportation is limited, to best provide access for care to those that need it most.

Local health departments are a valuable resource for community education and outreach. By partnering with local health departments, physicians are able to target at risk populations, including patients with low socioeconomic status. This partnership can provide education and facilitate connections with affordable local healthcare clinics and providers. Working with the local health department allows for the implementation of peer-based, culturally relevant education regarding nutrition and exercise paired with behavioral coaching and support. This approach significantly improved diabetes control, especially in low socioeconomic populations.^24^–^26^

## Rural Community Resources

### Available Food Sources

Community-based resources are essential to chronic disease development and prevention.^13^,^27^ One way to prevent disease is through promotion of healthy eating. Rural communities are unique in that there is ready access to locally grown fresh food. This can be obtained from local farmers or backyard gardens. In rural communities, the population density is less than in urban living, which allows for more available land to grow food. Physicians can aid in the promotion of locally grown food through education about the benefits of natural foods and working with community leaders on outreach programs promoting locally grown food. Additionally, in rural areas, people have easier access to local diary, eggs, and meat through local farmers. This availability allows for reduced transit time of food and fewer “food miles” resulting in more nutrient dense food with fewer preservatives.^28^

An additional benefit to rural living is reduced access to unhealthy fast food.^18^ In most rural communities in Kansas, very few fast food restaurants exist in the town or within a reasonable driving distance. This is beneficial in that studies have shown that the number of available fast food restaurants is correlated with BMI. These data suggest that food access plays a role in obesity and determinants of overall health. Limited access to fast food promotes cooking at home with less fried food and added sugars.

### Community Exercise

Obtaining adequate physical activity can be a challenge in any setting. One way that healthcare providers can aid patients in obtaining exercise is by writing an exercise prescription.^29^ Patients often do not know the level of recommended exercise or how best to implement an exercise program for the promotion of overall health. Exercise prescriptions, similar to any other prescription, explicitly written on script paper result in increased patient exercise and overall health.^29^ This method of healthcare delivery gives the patient specific instructions on the method and duration (i.e., 30 minutes of walking daily 5 times per week) and gives the patient a tangible objective for improving health.

Access to exercise equipment can be limited in rural areas with an absence of community-based gyms or exercise facilities. One option to increase access is to work with local school officials to allow the public access to school gyms and exercise equipment. This accessibility can be beneficial in that it allows families to exercise together and with other community members to promote overall health throughout the community. Previous studies in people with pre-diabetes have shown that resistance training at least twice a week improves fasting blood sugar levels and delays the development of diabetes.^24^

Physicians can engage community members to establish walking or biking paths to promote exercise. Community organizations, including churches and Veterans of Foreign Wars, are an outlet for community engagement and the promotion of health and disease prevention. Members of these organizations can engage in health promotion through organizing group exercise activities for both socialization and community outreach to benefit local charitable organizations. Community-based walks or runs can play an essential role in health promotion and community support. In addition, these sites of community gatherings can provide a means to screen for diabetes and distribute information about healthy lifestyle changes.

### Group Education Classes

Access to registered dieticians and diabetes educators in small communities can be limited.^29^ Practitioners can help with patient education through group education classes led by physicians, physician assistants, nurse practitioners, or other health care providers. In these sessions, it is important to address healthy eating patterns and counting carbohydrate content in foods. This approach empowers patients to learn about nutrient content in food and allows for improved dietary consumption. Additionally, these sessions can help the physician understand common barriers that patients face with healthy eating and facilitate group discussions on methods to overcome barriers. This atmosphere promotes a sense of community and provides participants with tools to live a healthy lifestyle. Group education sessions also can be a means to disseminate information about disease management and prevention of long-term complications, including retinopathy, nephropathy, neuropathy, and infection treatment and prevention.

### Weight Loss Programs

Weight loss options in rural areas are similar to urban areas, but tend to be smaller groups that meet less frequently or online-based alternatives.^29^ As in urban populations, the key to weight loss success depends less on the individual diet, but rather the individual adhering to the diet.^19^ Weight loss programs that incorporate education about healthy eating and nutrient content of food, increased exercise, and behavioral modifications result in greater weight loss and improved long-term maintenance of weight loss.^19^,^30^

Physicians can aid patients with weight loss by frequent monitoring of patient progress through weekly office weight checks, phone calls, or interactions through patient portals. Incorporating motivational interviewing in the initial office visits can lead to better health and weight loss maintenance, with visit frequency tapering over time. Determining a patient’s stage in the process is essential to the promotion of weight loss and will help to identify barriers to implement change.

### Community Outreach

Community outreach is a cornerstone to prevention and treatment of chronic disease.^13^ Physicians play an essential role in patient education and implementation of strategies to promote overall health in their communities. In rural communities, providers can work with local government officials to implement community wide health programs that focus on healthy eating, exercise, and community engagement. This outcome can be achieved by organizing walking groups to promote exercise, engagement with local famers and retailers to promote locally grown food, and working with the local media to provide education about healthy lifestyles.

Physicians can engage local schools to promote health during school hours, which often will translate to healthy living at home.^27^ By working to ensure healthy meals in schools, increased physical activity, and education about health promotion, physicians can create a healthier community. Additional outreach opportunities in rural communities include screening clinics and the distribution of health literature at community events, including local high school football and basketball games. This allows for the promotion of healthy living both within the community and in surrounding communities.

## Conclusions

Diabetes continues to have a significant negative overall impact on the health of Americans, especially here in Kansas. Physicians have numerous ways to engage patients to achieve a healthier lifestyle. Community outreach and a team-based approach to the treatment of diabetes are essential. By utilizing the resources in our local communities in a multidisciplinary approach, physicians will be better equipped to address the overall health of our patients. Physician advocacy is a key component to promote patient health and can serve not only as a means to engage local leaders in the promotion of population health, but also bring greater awareness to areas for improvement. By working together as a team within communities, practitioners are able to address patient needs and eliminate barriers to health promotion.

## References

[1] US Centers for Disease Control and Prevention National Diabetes Statistics Report.

[2] Ablah E, Dong F, Cupertino AP, Konda K, Johnston JA, Collins T (2013). Prevalence of diabetes and pre-diabetes in Kansas. Ethn Dis.

[3] Kansas Department of Health and Environment (2016). Kansas Diabetes and Prediabetes Facts.

[4] American Association of Clinical Endocrinologists. AACE Diabetes Resource Center http://outpatient.aace.com/type-2-diabetes/the-burden-of-diabetes.pdf.

[5] Dress B (2016). Reducing the burden of prediabetes and diabetes mellitus in the Kansas City metropolatin area: A call to action. Kansas City Medicine.

[6] US Agency for Healthcare Research and Quality. Healthcare Cost and Utilization Project (HCUP) (2005). http://www.ahrq.gov/research/data/hcup/index.html.

[7] Block G, Azar KM, Romanelli RJ (2016). Improving diet, activity and wellness in adults at risk of diabetes: Randomized controlled trial. Nutr Diabetes.

[8] Fowler MJ (2008). Microvascular and macrovascular complications of diabetes. Clin Diabetes.

[9] University of Kansas Medical Center (2012). KU Medical Center program offers diabetes management help for rural Kansas. KUMC News.

[10] Smetana GW, Abrahamson MJ, Rind DM (2016). Should we screen for type 2 diabetes?: Grand Rounds discussion from Beth Israel Deaconess Medical Center. Ann Intern Med.

[11] Mainous AG, Tanner RJ, Baker R (2016). Prediabetes diagnosis and treatment in primary care. J Am Board Fam Med.

[12] YMCA YMCA’s Diabetes Prevention Program.

[13] Hussein T, Kerrissey (2013). Using national networks to tackle chronic disease. Stanford Social Innovation Review.

[14] Philis-Tsimikas A, Gallo LC (2014). Implementing community-based diabetes programs: The Scripps Whittier Diabetes Institute experience. Curr Diab Rep.

[15] Block JP, Subramanian SV (2015). Moving beyond “Food Deserts”: Reorienting United States policies to reduce disparities in diet quality. PLoS Med.

[16] Creatore MI, Glazier RH, Moineddin R (2016). Association of neighborhood walkability with change in overweight, obesity, and diabetes. JAMA.

[17] Johns DJ, Hartmann-Boyce J, Jebb SA, Aveyard P (2014). Behavioural Weight Management Review Group. Diet or exercise interventions vs combined behavioral weight management programs: A systematic review and meta-analysis of direct comparisons. J Acad Nutr Diet.

[18] Li F, Harmer P, Cardinal BJ, Bosworth M, Johnson-Shelton D (2009). Obesity and the built environment: Does the density of neighborhood fast-food outlets matter?. Am J Health Promot.

[19] Johnston BC, Kanters S, Bandayrel K (2014). Comparison of weight loss among named diet programs in overweight and obese adults: A meta-analysis. JAMA.

[20] Garber AJ (2015). Anti-obesity pharmacotherapy and the potential for preventing progression from prediabetes to type 2 diabetes. Endocr Pract.

[21] Yanovski SZ, Yanovski JA (2014). Long-term drug treatment for obesity: A systematic and clinical review. JAMA.

[22] Sohler N, Matti-Orozco B, Young E (2016). Opportunistic screening for diabetes and prediabetes using hemoglobin A1c in an urban primary care setting. Endocr Pract.

[23] Lorig K, Ritter PL, Villa FJ, Armas J (2009). Community based peer-led diabetes self-management: A randomized trial. Diabetes Educ.

[24] Eikenberg JD, Savla J, Marinik EL (2016). Prediabetes phenotype influences improvements in glucose homeostasis with resistance training. PLoS One.

[25] Xiao H, Adams SR, Goler S (2015). Wellness coaching for people with prediabetes: A randomized encouragement trial to evaluate outreach methods at Kaiser Permanente, Northern California, 2013. Prev Chronic Dis.

[26] Thom DH, Ghorob A, Hessler D, De Vore D, Chen E, Bodenheimer TA (2013). Impact of peer health coaching on glycemic control in low-income patients with diabetes: A randomized controlled trial. Ann Fam Med.

[27] Perry CL, Luepker RV, Murray DM (1988). Parent involvement with children’s health promotion: The Minnesota Home Team. Am J Public Health.

[28] Hill H (2008). Food miles: Background and marketing.

[29] Butryn M, Webb V, Wadden T (2011). Behavioral treatment of obesity. Psychiatr Clin North Am.

[30] Kanat M, DeFronzo RA, Abdul-Ghani MA (2015). Treatment of prediabetes. World J Diabetes.

